# Metabolic Parameters Influence Brain Infarction and Outcome after Resection of Brain Metastases

**DOI:** 10.3390/cancers12051127

**Published:** 2020-04-30

**Authors:** Nicole Lange, Julia Urich, Melanie Barz, Kaywan Aftahy, Arthur Wagner, Lucia Albers, Stefanie Bette, Benedikt Wiestler, Martin Bretschneider, Bernhard Meyer, Jens Gempt

**Affiliations:** 1 Department of Neurosurgery, Klinikum rechts der Isar, School of Medicine, Technical University Munich, 81675 Munich, Germany; julia.u0203@gmail.com (J.U.); melanie.barz@tum.de (M.B.); kaywan.aftahy@tum.de (K.A.); arthur.wagner@tum.de (A.W.); lucia.albers@tum.de (L.A.); bernhard.meyer@tum.de (B.M.); jens.gempt@tum.de (J.G.); 2 Department of Neuroradiology, Klinikum rechts der Isar, School of Medicine, Technical University Munich, 81675 Munich, Germany; Stefanie.bette@tum.de (S.B.); benedikt.wiestler@tum.de (B.W.); 3 Department of Diagnostic and Interventional Radiology, Universitätsklinikum Augsburg, 86156 Augsburg, Germany; 4 Department of Anesthesiology, Klinikum rechts der Isar, School of Medicine, Technical University Munich, 81675 Munich, Germany; martin.bretschneider@tum.de

**Keywords:** postoperative infarction, ischemia, brain metastases, outcome

## Abstract

Perioperative infarction in brain tumor surgery occurs in about 30–80% of cases and is strongly associated with poor patient outcomes and longer hospital stays. Risk factors contributing to postoperative brain infarction should be assessed. We retrospectively included all patients who underwent surgery for brain metastases between January 2015 and December 2017. Hemodynamic parameters were analyzed and then correlated to postoperative infarct volume and overall survival. Of 249 patients who underwent biopsy or resection of brain metastases during that time, we included 234 consecutive patients in this study. In total, 172/249 patients showed ischemic changes in postoperative magnet resonance imaging (MRI) (73%). Independent risk factors for postoperative brain infarction were perioperative blood loss (rho 0.189, *p* = 0.00587), blood glucose concentration (rho 0.206, *p* = 0.00358), blood lactate concentration (rho 0.176; *p* = 0.0136) and cumulative time of reduced PaCO_2_ (rho −0.142; *p* = 0.0445). Predictors for reduced overall survival were blood lactate (*p* = 0.007) and blood glucose levels (*p* = 0.032). Other hemodynamic parameters influenced neither infarct volume, nor overall survival. Intraoperative elevated lactate and glucose levels are independently associated with postoperative brain infarction in surgery of brain metastases. Furthermore, they might predict reduced overall survival after surgery. Blood loss during surgery also leads to more cerebral ischemic changes. Close perioperative monitoring of metabolism might reduce those complications.

## 1. Introduction

Perioperative infarction in brain tumor surgery occurs in about 30–80% of the cases and may lead to persistent neurological deficits and a delay of recovery [1,2,3,4]. Infarct volume in glioblastoma surgery has proven to be an independent prognostic factor, as postoperative neurological deficits have been proven to be associated with decreased overall survival and lower quality of life [5,6]. Perioperative ischemia leading to hypoxic tissue might also induce tumor growth and contribute to tumor aggressiveness [7,8,9]. Recent studies revealed intraoperative diastolic blood pressure, fluid balance and length of surgery as independent factors for development of postoperative ischemia [10]. Mean intraoperative diastolic blood pressure and middle arterial blood pressure were significant prognostic factors in a multivariate analysis in patients with glioma surgery. Data analyzing brain infarction after resection of brain metastases are rare.

Therefore, the aim of this study was to analyze hemodynamic parameters in patients with surgery of brain metastases and then correlate them to postoperative infarct volume and overall survival.

## 2. Materials and Methods

### 2.1. Patient Population

Retrospectively, 243 patients (124 female, 119 male) who underwent surgery for brain metastases between January 2015 and December 2017 were included. There were no exclusion criteria, apart from inconclusive histopathology or absence of perioperative documentation of hemodynamic parameters.

For all patients, early postoperative MRI (within 72 h postoperative, including diffusion-weighted images) and documentation sheets of pre-/intra- and postoperative hemodynamics were available.

The following data were assessed for every patient: clinical data including past medical history of high blood pressure, diabetes, peripheral arterial occlusive disease (PAOD), previous thromboembolic events, smoking status, pre- and postoperative Karnofsky Performance Status Scale (KPS), date of initial tumor diagnosis, as well as of surgery, histopathological workup, systemic metastases, date of death or last contact and dates of recurrence. Ethical approval was obtained by the local ethics commitee (reference number 160/15S). As a retrospective analysis, patient consent was not necessary.

### 2.2. Anesthesia and Hemodynamic Parameters

General anesthesia was performed according to institutional standards with propofol and remifentanil. Directly before skin incision, perioperative antibiotic prophylaxis was applied (usually cefuroxime 1.5 g) and mannitol was given (in a dose of 20 g) to further reduce intracranial pressure. Fluid resuscitation was managed with lactated ringer solution and transfusion of blood products was performed following institutional standards. After surgery, prompt extubation was strived for whenever possible and patients were then transferred to post-anesthesia care units to be monitored for one night.

Hemodynamic parameters, which were prospectively documented before, during and after surgery, were collected in every patient. Those were in detail: duration of surgery, blood loss, requirement of catecholamine, blood pressure as measured every 5 min intraoperatively (an arterial line was established in every patient), oxygen saturation, temperature during surgery, fluid substitution and transfusion of blood products, as well as blood gas analyses whenever measured. Furthermore, the cumulative time of reduced blood pressure (<20% of initial BP) and paCO2 (<30 mmHg) was calculated. The minimum hemoglobin concentration (Hb) was assessed in all patients. Blood glucose and lactate levels were documented in every patient directly before the start of surgery with blood gas analysis. Routine perioperative administration of steroids was abandoned in our clinic and no additional steroids were given which could have influenced blood glucose levels.

### 2.3. Statistical Data Analysis

Statistical analyses, including descriptive data analyses, were performed using 3.6.2 (R Core Team, https://www.r-project.org/). Associations between all assessed variables were analyzed using chi-square tests. To identify possible risk factors for outcome changes, logistic regression analysis was done. For all analyses, a difference with an error probability of less than 0.05 was considered statistically significant. Descriptive statistics for demographic variables were generated with means and SDs or medians with interquartile ranges as appropriate. Overall survival analyses were performed using Kaplan–Meier estimates for univariate analysis and a Cox regression proportional hazards model for multivariate analysis. For associations between hemodynamic parameters and postoperative infarct volume, Fisher’s exact test was used; sensitivity and specificity were calculated for the features (stratified based on the median). Spearman’s correlation coefficient was used to assess the relation between hemodynamics and ischemia volume. To account for type I error inflation, the false discovery rate method proposed by Benjamini and Hochberg was employed. Furthermore, bootstrapping (repeated 1000 times) was performed to estimate a 95% confidence interval around the correlation coefficient rho.

### 2.4. Magnetic Resonance Imaging and Image Analysis

MRI scans were performed with a 3 Tesla MRI scanner (Philips Achieva/Ingenia, Philips Medical Systems, Andover, The Netherlands B.V) or Siemens Verio (Siemens Healthcare, Erlangen, Germany). Protocols included T2 weighted fluid-attenuated inversion recovery (FLAIR) and T1 weighted sequences with and without contrast agent. Postoperative MRI scans further included diffusion-weighted images (DWI).

Image analysis was performed by a neuroradiologist and by a neurosurgeon in consensus, blinded to hemodynamic parameters. Postoperative changes as well as methemoglobin were excluded by assessing (fluid attenuated inversion recovery) FLAIR images and T1w images without contrast agent, as was described before [1,2,3,5]. Manual segmentation of postoperative ischemia was performed by an experienced neurosurgeon (NL, 5 years of experience) blinded to hemodynamic parameters and controlled by an experienced neuroradiologist (BW, 8 years of experience). Semiautomatic segmentation software (Origin^®^ 3.1 cranial planning software, Brainlab AG, Munich, Germany) was used as described before. In addition, the volumes of the pre- and postoperative contrast-enhancing tumor parts were assessed using manual segmentation.

## 3. Results

### 3.1. Patient Population

Patient characteristics are presented in Table 1.

**Figure 1:**
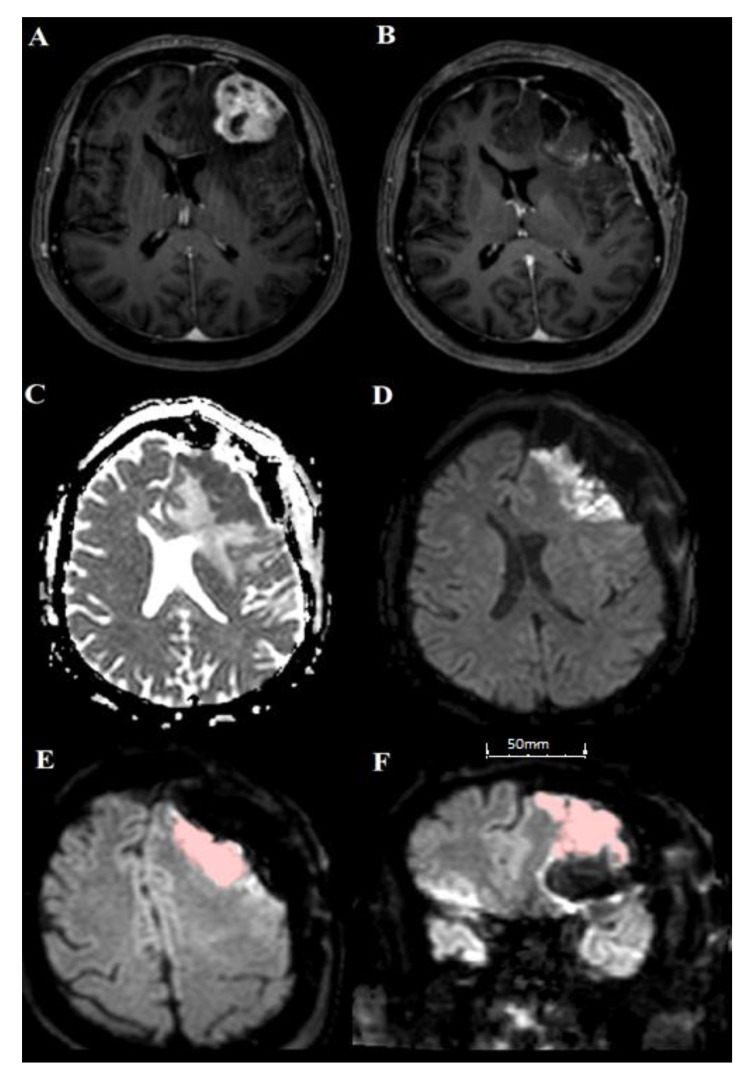
Example of one patient (70-year-old female with breast cancer) with left frontal metastasis. (**A**,**B**) show T1 contrast-enhanced images to compare preoperative (left) and postoperative (right). (**C**,**D**) show left frontal infarction with hyperintensity in b-1000 images (**D**) and corresponding hypointensity in ADC (**C**). (**E**,**F**) show an example of semiautomatic volumetric measurement of the postoperative infarct region, axial (**E**) and coronar (**F**).

Between January 2015 and December 2017, 243 consecutive patients (124 female, 119 male) who underwent resection of histopathologically proven brain metastases were retrospectively included. In that time interval, we recorded 249 patients who underwent biopsy or resection due to an initial diagnosis of brain metastases. The mean age at the date of surgery was 61 years (range 18–86 years). Due to recurrent disease, 7.3% underwent surgery. At the time of surgery, 54.3% had systemic metastases. The median tumor size was 1.2 cm^3^ (interquartile range 0.25–3.75). Overall, 22% of the patients showed hemorrhage metastases. All metastases were resected completely (removal of contrast-enhancing tissue). Tumor entities are presented in Table 2 and Figure 1. The most frequent primary tumors were lung and breast cancer and malignant melanoma [11,12]. Localization of metastases was: frontal (35%), parietal (24%), temporal (12%), occipital (10%), cerebellar (19%) and in multiple lobes in 2% of cases.

The median KPS Score was 80 (interquartile range (IQR) 70–90) pre- and postoperative. Regarding comorbidities, arterial hypertension was recorded in 35.5%, diabetes in 10.5%, previous thromboembolic events in 8.7%, and peripheral arterial occlusive disease (PAOD) in 2.2% of cases. Of the total, 31% were smokers. The mean duration of surgery was 147 min. Mean blood loss was 363.7 mL. The median overall survival was 1507 days/50 months (range 6–1906 days); progression-free survival was 209 days/seven months (range 1–1898 days).

Patients presented with the following symptoms: (hemi) -paresis (M < 4/5) in 47 patients, (hemi) -paresis (M > 4/5) in 16 patients, moderate aphasia in 16 patients, severe aphasia in four patients, and vision field impairments in six patients.

### 3.2. Postoperative Ischemia

Volumetric measurements of postoperative ischemic changes are also summarized in Table 1. The median postoperative infarct volume was 1.1 cm^3^ (interquartile range 0–3.7). In total, 172/249 patients showed ischemic changes in postoperative MRI (73%). Figure 1 shows an example of a volumetric measurement of postoperative ischemia.

Regarding neurological outcomes, 9.3% of the patients with visible infarction on postoperative MRI (16/172) showed new neurological deficits, which were persistent over more than 3 months. Deficits were paresis M ≥ 4/5 in seven patients, paresis M < 4/5 in seven patients, and aphasia in two patients. Interestingly, within the patient collective without postoperative infarction, only 3.2% showed persistent new deficits (2/62; one case of mild paresis, one case of severe paresis). The mean postoperative KPS was 74 in the infarction-group versus 79 in the group without visible infarction on postoperative MRI.

**Figure 2:**
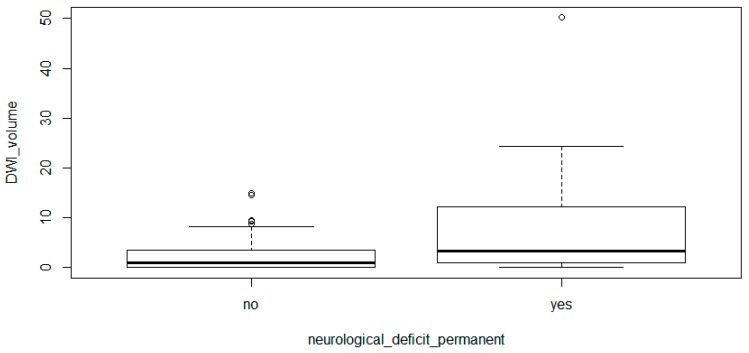
Comparison of patients with permanent (lasting more than 3 months) neurological deficits to patients with transient or no neurological deficits and their postoperative infarct volumes. Patients with new permanent deficits showed significantly higher infarct volumes.

Infarction volume significantly correlated with new postoperative neurological deficits (*p* = 0.006, 95% CI [−5.596, −0.470]). This is illustrated in Figure 2.

### 3.3. Correlation between Hemodynamic Parameters and Infarct Volumes

To assess correlations between hemodynamics and infarct volume, Spearman’s correlation coefficient was calculated for all parameters. To adjust for type I error inflation, we performed false-discovery rate correction with the Benjamini–Hochberg procedure (FDR = 0.15). Analysis revealed that blood loss (rho 0.189, *p* = 0.00587), blood glucose concentration (rho 0.206, *p* = 0.00358), blood lactate concentration (rho 0.176; *p* = 0.0136) and cumulative time of reduced PaCO_2_ (rho −0.142; *p* = 0.0445) are significantly associated with higher infarct volumes (Table 3).

Other hemodynamic parameters showed only borderline significance (cumulative time of reduced heart rate: *p*= 0.0706; mean systolic blood pressure during surgery: *p* = 0.0844; oxygen saturation: *p* = 0.0719; mean heart rate *p* = 0.0937).

No significance regarding postoperative infarction was recorded for blood-pH levels, transfusion of blood products, mean arterial blood pressure during surgery, mean hemoglobin concentration, length of surgery or use of catecholamine during surgery.

Furthermore, no significant correlation was observed for any clinical patient characteristics, such as co-morbidities, smoking status, recurrent tumors, hemorrhage metastases, or KPS.

### 3.4. Overall Survival

Univariate and multivariate survival analyses were calculated for all patients. Univariate survival analysis using Kaplan–Meier estimates (log rank) for survival from initial diagnosis of tumor revealed the following parameters as significant prognostic factors: blood lactate concentration (*p* = 0.023), cumulative time of reduced diastolic blood pressure during surgery (*p* = 0.070), reduced mean arterial pressure during surgery (*p* = 0.012), reduced oxygen saturation (*p* = 0.027).

**Figure 3:**
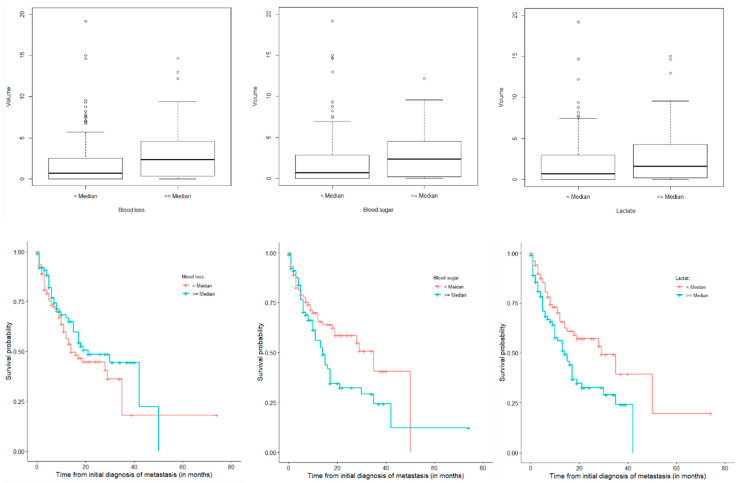
Box plots (dichotomized by the respective median) and Kaplan–Meier estimates of blood loss, blood glucose and lactate levels. Infarct volume.

Analysis for survival from surgery confirmed blood lactate (*p* = 0.007) as significant (see Figure 3). Additionally, blood glucose levels also showed statistical significance (*p* = 0.032). Blood loss was not significant in this model.

Multivariate survival analysis was performed using a Cox regression proportional hazard model. This model did not confirm any of the above-mentioned parameters as significant prognostic factors for reduced survival, with the exception of blood lactate (HR 1.61, 95% CI [1.03–2.51], *p* = 0.038). Interestingly, known factors for reduced overall survival in primary brain tumors, such as age and KPS or any co-morbidity seem not to be relevant prognostic factors for patients with brain metastases.

Concerning early, possibly surgery-related deaths, only three patients died within the first 30 days after surgery. This included two patients who showed recurrent bleeding into the resection cavity and were systemically progressive, so a decision was made for best supportive care. One patient suffered pneumonia due to aspergillus during postoperative whole-brain radiation.

## 4. Discussion

Hemodynamic parameters have proven to be of significance concerning infarct volumes after glioma surgery. In particular, the mean intraoperative diastolic blood pressure seems to be independently associated with overall survival and postoperative ischemic volumes [10].

This analysis of a patient collective, having undergone resection of brain metastases, did not confirm the relevance of hemodynamic parameters, as the mean intraoperative diastolic blood pressure only reached borderline significance. It is probable that the higher diversity in disease characteristics in this patient collective, and the greater relevance of the underlying cancer, compared to glioma patients, put hemodynamics into the background.

Interestingly, blood glucose levels, blood lactate levels and blood loss were independently associated with higher postoperative infarct volumes.

Survival analyses confirmed blood lactate and blood glucose levels as significant predictors for reduced overall survival after surgery for brain metastases. Hemodynamics did not significantly influence survival.

Survival analyses in this study did not take into account patients’ systemic tumor load or metabolic parameters, as the cohort was almost equally divided regarding the presence (54%) or absence (46%) of systemic metastases.

These results suggest that the maintenance of a sufficient metabolism is of great relevance to the postoperative prevention of ischemic changes.

Studies show that hyperglycolysis, followed by elevated lactate concentrations in the penumbra zone of stroke patients, leads to more brain infarctions [13]. Elevated admission glucose levels were associated with brain infarction and with a higher 5-year mortality [14].

Analyses of 436 patients undergoing craniotomies for various reasons revealed that elevated serum lactate levels during surgery were associated with new neurological deficits (odds ratio 2.11) and longer hospital stays (20% less likely to be discharged on a given day). It did not lead to more systemic complications such as myocardial infarction or mortality [15]. This poses the question of whether parts of the new neurological deficits could be explained by lactate-induced ischemia. In our patient collective, only 15 patients showed permanent new neurological deficits after surgery (6.4%). (Eight patients suffered severe deficits, such as motor weakness <4/5, seven patients suffered non-severe deficits, such as motor weakness >4/5 or vision field impairment).

Elevated blood glucose and lactate levels in critically ill patients have both been intensively studied, hypothesizing that they may be increased due to sympathetic activation and are known to be independent risk factors for worse outcomes [16,17]. In patients with an acute subarachnoid hemorrhage, they were associated with delayed cerebral ischemia-related infarction and poor outcomes as measured by a modified Rankin Scale at 3 months after bleeding [18,19,20].

Blood loss was not a significant factor for overall survival in multivariate analysis, but seemed to significantly correlate with postoperative infarct volumes. Haemodynamic responses to acute blood loss also lead to sympathetic vasoconstrictor drive, being in line with elevated glucose and lactate levels [21].

Hypocapnia causes cerebral vasoconstriction and is known as a poor prognostic factor during subarachnoidal hemorrhages (aSAH) and ischemic strokes [22,23]. In this study, hypocapnia during surgery also significantly correlated negatively with postoperative brain infarction.

To our knowledge, these parameters have not yet been assessed during elective brain surgery.

Perioperative infarction in brain tumor surgery occurs in about 30–80% of the cases and may lead to persistent neurological deficits and a delay of recovery [1,2,3,4]. Infarct volume in glioblastoma surgery has proven to be an independent prognostic factor, as postoperative neurological deficits have been proven to be associated with decreased overall survival and lower quality of life [5,6]. This stands in line with the correlation of neurological deficits and infarct volume, as shown earlier and confirmed by this work [2]. Perioperative ischemia leading to hypoxic tissue might also induce tumor growth and contribute to tumor aggressiveness [7,8,9]. Therefore, understanding the contributing factors of postoperative infarction in order to address them in future surgeries is of great importance for patient outcomes.

Our results suggest that close monitoring of metabolism during brain surgery might reduce ischemia-related complications and improve patient outcomes.

## 5. Conclusions

During resection of brain metastases, intraoperatively elevated lactate and glucose levels, as well as blood loss during surgery, are independently associated with volumes of postoperative brain infarction. Blood lactate might also be an independent prognostic factor for overall survival. Therefore, close monitoring of these parameters during surgery is recommended to reduce the volume of postoperative infarctions. Prospective studies are necessary to confirm the results of this retrospective study.

## Figures and Tables

**Table 1:** Patient characteristics.

Age at Date of Surgery (*n* = 234)	Mean 61.3 (Range 18–86) Years
Age at date of initial diagnosis (*n* = 213)	Mean 58.1 years
Sex, female	124/234 (53.0%)
Recurrent disease	17/233 (7.3%)
KPS preoperative (*n* = 232)	80 (20–100) %
KPS postoperative (*n* = 234)	80 (0–100) %
Death during FU	99/234 (42.3%)
Arterial hypertension	81/228 (35.5%)
Diabetes	24/229 (10.5%)
Previous thromboembolic events	20/229 (8.7%)
PAOD	5/228 (2.2%)
Smoker	71/229 (31.0%)
Postoperative infarct volume (*n* = 213)	Median 1.1 (IQR 0–3–7) cm^3^
OP Time (*n* = 225)	Mean 147.1 (SD 62.3) min
Blood loss (*n* = 231)	Mean 363.7 (SD 349.4) mL

KPS = karnofsky performance status; FU = folow up; PAOD = peripheral arterial occlusive disease.

**Table 2:** Distribution of tumor entity.

Tumor Entity	Tumor Entity-Subgroup	No. Patients	%
	lung	75	32.1
	breast	37	15.8
	malignant melanoma	35	15.0
gastrointestinal	rectum	8	3.4
	colon	6	2.6
	AEG	3	1.3
	pancreas	2	0.9
	gastric	1	0.4
	sigma	1	0.4
urogenital	renal	8	3.4
	bladder	4	1.7
men	prostate	4	1.7
	testicle	7	3.0
women	ovarial	7	3.0
	endometric	1	0.4
	hepatocellular	4	1.7
	parotid	4	1.7
	thyroid	2	0.9
	skin	3	1.3
	MPNST	2	0.9
	ewing sarcoma	1	0.4
	choroidal melanoma	1	0.4
	plasmocytoma	1	0.4
	unknown	17	7.3

**Table 3:** Correlations of hemodynamic parameters and infarct volume.

Parameter	*p*	(i/m) × *q*; *q* = 0.15	Spearman’s Coefficient with FDR Correction
**blood glucose (mg/dL)**	0.00358315	0.0046875	0.20659276
**blood loss (mL)**	0.00587021	0.009375	0.18951449
**lactate mg/dL (mmol/L)**	0.01368411	0.0140625	0.17630288
**PaCO_2_ (mmHg)**	0.04454785	0.01875	−0.14293733
**heart rate SD**	0.07068349	0.0234375	0.12468846
**oxygen saturation SD**	0.07193419	0.028125	0.12414285
**systolic blood pressure, mean**	0.08442738	0.0328125	0.11907649
**heart rate, mean**	0.0937836	0.0375	0.11566422
